# Monoamine Oxidase A (*MAOA*) Gene and Personality Traits from Late Adolescence through Early Adulthood: A Latent Variable Investigation

**DOI:** 10.3389/fpsyg.2017.01736

**Published:** 2017-10-11

**Authors:** Man K. Xu, Darya Gaysina, Roula Tsonaka, Alexandre J. S. Morin, Tim J. Croudace, Jennifer H. Barnett, Jeanine Houwing-Duistermaat, Marcus Richards, Peter B. Jones, the LHA Genetics Group

**Affiliations:** ^1^Faculty of Psychology and Educational Sciences, Welten Institute, Open University of the Netherlands, Heerlen, Netherlands; ^2^Department of Medical Statistics and Bioinformatics, Leiden University Medical Centre, Leiden, Netherlands; ^3^Department of Psychiatry, University of Cambridge, Cambridge, United Kingdom; ^4^Department of Psychology, Education, and Child Studies, Erasmus University Rotterdam, Rotterdam, Netherlands; ^5^EDGE Lab, School of Psychology, University of Sussex, Brighton, United Kingdom; ^6^Substantive-Methodological Synergy Research Laboratory, Department of Psychology, Concordia University, Montreal, QC, Canada; ^7^School of Nursing and Health Sciences, University of Dundee, Dundee, United Kingdom; ^8^Cambridge Cognition, Cambridge, United Kingdom; ^9^MRC Unit for Lifelong Health and Ageing at UCL, London, United Kingdom

**Keywords:** latent variable, *MAOA*, personality, development, adolescents

## Abstract

Very few molecular genetic studies of personality traits have used longitudinal phenotypic data, therefore molecular basis for developmental change and stability of personality remains to be explored. We examined the role of the monoamine oxidase A gene (*MAOA*) on extraversion and neuroticism from adolescence to adulthood, using modern latent variable methods. A sample of 1,160 male and 1,180 female participants with complete genotyping data was drawn from a British national birth cohort, the MRC National Survey of Health and Development (NSHD). The predictor variable was based on a latent variable representing genetic variations of the *MAOA* gene measured by three SNPs (rs3788862, rs5906957, and rs979606). Latent phenotype variables were constructed using psychometric methods to represent cross-sectional and longitudinal phenotypes of extraversion and neuroticism measured at ages 16 and 26. In males, the *MAOA* genetic latent variable (AAG) was associated with lower extraversion score at age 16 (β = −0.167; CI: −0.289, −0.045; *p* = 0.007, FDRp = 0.042), as well as greater increase in extraversion score from 16 to 26 years (β = 0.197; CI: 0.067, 0.328; *p* = 0.003, FDRp = 0.036). No genetic association was found for neuroticism after adjustment for multiple testing. Although, we did not find statistically significant associations after multiple testing correction in females, this result needs to be interpreted with caution due to issues related to x-inactivation in females. The latent variable method is an effective way of modeling phenotype- and genetic-based variances and may therefore improve the methodology of molecular genetic studies of complex psychological traits.

## Introduction

Personality traits, such as neuroticism and extraversion, are relatively stable during adulthood (Roberts et al., 2008); however their mean levels are subject to change from adolescence through early adulthood (Roberts and DelVecchio, 2000; Soto et al., 2011; Specht et al., 2011). It is known that personality traits are heritable polygenic traits (Bouchard and Loehlin, 2001; Ebstein, 2006; Pilia et al., 2006; Benjamin et al., 2008; Vernon et al., 2008; Distel et al., 2009), with quantitative behavioral genetic studies suggesting that genetic factors can contribute to stability and age-related changes (Wray et al., 2007; Bleidorn et al., 2009; Kandler et al., 2012). However, no previous studies have investigated associations between specific genetic variation and personality traits from a developmental perspective based on repeated measures of personality traits in adolescence and in early adulthood.

Personality traits have been viewed as endophenotypes for different psychiatric disorders (Terracciano et al., 2010). Indeed, neurobiological correlates, as well as genetic factors, common for personality traits and psychiatric disorders have been found (Foster and MacQueen, 2008; Luciano et al., 2012; Gale et al., 2016; Okbay et al., 2016). Therefore, candidate genes implicated in psychopathology can also be involved in the development of personality traits.

Recent molecular genetic studies have provided multiple lines of evidence supporting the role of the monoamine oxidase A gene (*MAOA*) in various psychopathologies in adults and children, including antisocial behavior (Fergusson et al., 2011; Ouellet-Morin et al., 2016), autism spectrum disorder (ASD; Tassone et al., 2011; Verma et al., 2014), and impulsivity (Kinnally et al., 2009; Enoch et al., 2010). *MAOA* is primarily expressed in catecholaminergic neurons in the human brain (Thorpe et al., 1987), and it preferentially metabolizes serotonin and norepinephrine (Arai et al., 1997). This key function of the *MAOA* in the central nervous system (CNS) provides the strong rationale for studying *MAOA* gene in personality and other complex psychological traits.

Studies using *MAOA* knockout mice models have established that *MAOA* deficiency leads to neurochemical imbalances, which culminates in neuroanatomical abnormalities such as reduced thickness of corpus callosum, increased dendritic arborization of pyramidal neurons in the prefrontal cortex and disrupted microarchitecture of cerebellum (Bortolato et al., 2013). Human studies have also provided some evidence for the role of common genetic variants in the MAOA function. Specifically, a variable repeat (VNTR) in the 5-flanking region of the *MAOA* gene demonstrated allele-specific variation in promoter activity in an *in vitro* assay system (Sabol et al., 1998). Another study (Jansson et al., 2005) showed that in females the C/C and C/T genotypes of rs979605 (also corresponds to A/A and A/G genotypes of rs979606 in our study) were associated with a significant decrease in thrombocyte-MAO (Trbc-MAO) enzyme activity (expressed as nmoles of 2-phenylethylamine oxidized per minute and per 1010 platelets; Pedersen et al., 1993). In addition, four-SNP haplotype (rs1801291, rs979605, rs6323, rs388863) was associated with Trbc-MAO activity. This association may reflect MAOA activity in the brain. Alternatively, it is possible that cis-acting regulatory elements within *MAOA* gene can affect MAOB platelets expression. Another plausible explanation could be that *MAOA* single nucleotide polymorphisms (SNPs) affected by methylation lead to changes in the expression pattern.

A number of common SNPs were also shown to contribute to allelic mRNA expression in human brain (Pinsonneault et al., 2006). Moreover, there is also evidence for the CNS structural and functional changes related to the *MAOA* common genetic variants (Manuck et al., 2000; Ducci et al., 2006; Meyer et al., 2006).

To date, there have been few association studies of the *MAOA* gene and personality traits, with the results being largely inconsistent, likely due to small sample sizes (Jorm et al., 1997, 2000; Eley et al., 2003). Although, there are recent well-powered large-scale genome-wide association studies (GWAS) of personality traits (Vinkhuyzen et al., 2012; de Moor et al., 2015; Okbay et al., 2016; van den Berg et al., 2016; Lo et al., 2017), the *MAOA* gene is usually not included in these studies because it is located on the X-chromosome.

Several studies have reported a sexual dimorphic effect of *MAOA* gene in behavioral traits (Verma et al., 2014), which is indicative of the underlying sexual dimorphism in the regulation of MAOA enzyme (Wu et al., 2009). Moreover, as MAOA activity levels increase with age (Breakefield et al., 1980; Hotamisligil and Breakefield, 1991), the effect of the *MAOA* gene can be more pronounced at later developmental stages. For example, one study showed that differential associations between the *MAOA* genotype, neural response and stimuli of social rejection were present among adult females, but not among adolescent females (Sebastian et al., 2010). This observation suggests that as the neural circuits associated with higher mental function continue to develop through adolescence, associations between the *MAOA* gene and psychological phenotypes, including personality traits, may change during transition from adolescence to adulthood.

In the present study, we investigated the effects of the *MAOA* genetic variants on extraversion and neuroticism from adolescence (age 16) through early adulthood (age 26), using data from the MRC National Survey of Health and Development (NSHD), also known as the British 1946 birth cohort. We hypothesized that the effects of the *MAOA* gene on personality traits would change through maturation, and may differ between males and females.

Genetic associations are typically estimated from univariate analysis based on one SNP or several SNPs within a gene region that are analyzed separately. It has been proposed that summary latent genetic variables can better capture genetic variance (Smyrnis et al., 2009; Tsonaka et al., 2012; Bentley et al., 2013). In order to more robustly assess the relationship between *MAOA* genetic variation and personality traits, a latent genetic variable was created based on three *MAOA* SNPs: rs3788862, rs5906957, and rs979606.

We also used psychometric methods to model the personality phenotypes at ages 16 and 26 (i.e., cross-sectional model) and the age-related changes from 16 to 26 in the phenotypes (i.e., longitudinal model). The utilization of psychometric latent variable methods is advantageous in that personality phenotypes constructed this way are corrected for measurement errors, thus providing more accurate estimations of genetic effects (Xu et al., 2015). This is particularly the case for longitudinal phenotypes, which are especially prone to measurement errors when difference scores are derived from repeated data (Thomas and Zumbo, 2012; Kisbu-Sakarya et al., 2013). Specifically, we use latent difference models (McArdle and Hamagami, 2001) to estimate the age-related changes of extraversion and neuroticism from age 16 to age 26.

Since the *MAOA* gene is located on the X chromosome (Xp11.23), and there is evidence for incomplete inactivation of X-chromosome in females (Berletch et al., 2011), all analyses were performed separately for males (XY karyotype) and females (XX karyotype), in line with other genetic association studies of *MAOA* (Eley et al., 2003; Liu et al., 2011). Results specific for females were interpreted with caution.

## Methods

### Sample

The NSHD is a socially stratified birth cohort of 5362 individuals of a white Caucasian background, who have been followed up since their birth in 1946 with multiple data collections across the life course (Wadsworth et al., 2003). At age 53, the blood samples were collected for DNA extraction and genetic analyses. Those interviewed at the age of 53 (*n* = 3035) were, in most respects, representative of the national population of that age born in Britain (Wadsworth et al., 2003). Almost all participants (*n* = 2,900, 96% of the available sample) provided a blood sample. Ethical approval for this research was obtained from the North Thames Multi-Centre Research Ethics Committee and from relevant local research ethics committees in the survey areas. Informed consent was given by all the respondents. All analyses were performed using Mplus 7.4, with the WLSMV estimator and the theta parameterisation (Muthén and Muthén, 2015).

### Personality trait measures

At ages 16 and 26 years, neuroticism and extraversion were assessed with six questions for each trait using the short from of the Maudsley Personality Inventory (MPI; Eysenck, 1958). All items had a binary “yes” (1) or “no” (0) response category.

### SNP selection and genotyping

DNA was extracted and purified from whole blood using the Puregene DNA Isolation Kit (Flowgen, Leicestershire, UK) according to the manufacturer's protocol. The three *MAOA* SNPs, rs3788862, rs5906957, and rs979606 (Table 1), were typed using the KASPar system by KBioscience, UK (www.kbioscience.co.uk). These SNPs were selected using a Tagger implementation of the Haploview programme 9 (Barrett et al., 2005) to provide adequate coverage of the *MAOA* gene region. Two of these SNPs (rs979606 and rs3788862), or other SNPs mentioned earlier that are in high/complete LD with them, have been previously reported to be associated with *MAOA* functional activity (Hsu et al., 1995; Jansson et al., 2005).

**Table 1:** Descriptive for *MAOA* SNPs allele, genotype and LD structure in males and females.

**Snp**	**Chromosome location**	**Alleles (minor)**	***n***	**MAF**		***n***	**MAF**	**HWE *p*-value**
			**Males**			**Females**		
rs3788862	43402308	A/G	1,240	0.300		1,258	0.280	0.400
rs5906957	43432254	A/G	1,244	0.240		1,260	0.220	0.870
rs979606	43486086	G/A	1,240	0.300		1,257	0.290	0.130
			**LD structure**					
			**rs3788862**	**rs5906957**	**rs979606**	**rs3788862**	**rs5906957**	**rs979606**
			**Males**			**Females**		
rs3788862	43402308	A/G	1	0.680	0.750	1	0.700	0.790
rs5906957	43432254	A/G		1	0.520		1	0.560
rs979606	43486086	G/A			1			1

*SNP, Single Nucleotide Polymorphisms; MAF, Minor allele frequency; HWE, Hardy-Weinberg equilibrium*.

For SNPs rs3788862 and rs5906957, in males A allele (the minor allele) was coded as 0 and G allele as 1; in females genotype AA was coded as 0, AG as 1, and GG as 2. For SNP rs979606, in males G allele (the minor allele) was coded as 0 and A allele as 1; in females genotype GG was coded as 0, AG as 1, and AA as 2. The three SNPs were used as binary (in males) or ordinal (in females) indicator variables for psychometrically modeling the latent genetic variable (Table 2). As such, positive factor loadings indicate the number of minor allele SNP contributed positively to the latent genetic factor whereas a negative factor loading indicate a negative direction.

**Table 2:** Descriptives and factor loadings for extraversion and neuroticism at ages 16 and 26 and *MAOA* SNPs.

	**Male**					**Female**						
		**Yes**		**No**			**Yes**		**No**			
	**Loadings**	**%**	**Count**	**%**	**Count**	**Loadings**	**%**	**Count**	**%**	**Count**		
**EXTRAVERSION 16**												
Are you happiest when you get involved in some project which calls for rapid action?	0.417	0.306	279	0.694	634	0.392	0.437	405	0.563	522		
Do you usually take the initiative in making new friends?	0.428	0.340	294	0.660	571	0.520	0.291	266	0.709	647		
Are you inclined to be quick and sure in your actions?	0.626	0.305	255	0.695	580	0.660	0.451	385	0.549	468		
Would you rate yourself as a lively individual?	0.689	0.172	151	0.828	727	0.742	0.218	193	0.782	692		
Would you be very unhappy if you were prevented from making numerous social contacts?	0.286	0.318	299	0.682	641	0.296	0.332	329	0.668	663		
Do you prefer action to planning for action?	0.239	0.321	287	0.679	608	0.284	0.358	335	0.642	602		
**NEUROTICISM 16**												
Do you sometimes feel happy, sometimes depressed, without any apparent reason?	0.676	0.547	538	0.453	446	0.620	0.224	234	0.776	809		
Does your mind often wander while you are trying to concentrate?	0.520	0.456	452	0.544	539	0.571	0.355	365	0.645	664		
Are you frequently 'lost in thought' even when supposed to be taking part in a conversation?	0.518	0.712	694	0.288	281	0.576	0.637	649	0.363	370		
Are you sometimes bubbling over with energy and sometimes very sluggish?	0.571	0.494	476	0.506	487	0.549	0.340	344	0.660	668		
Are you inclined to be moody?	0.808	0.627	608	0.373	361	0.859	0.504	502	0.496	494		
Do you have frequent ups and downs in mood either with or without apparent cause?	0.879	0.649	628	0.351	340	0.885	0.427	430	0.573	576		
**EXTRAVERSION 26**												
Are you happiest when you get involved in some project that calls for rapid action?	0.514	0.200	207	0.800	826	0.447	0.322	346	0.678	728		
Do you usually take the initiative in making new friends?	0.527	0.405	403	0.595	591	0.581	0.397	421	0.603	640		
Are you inclined to be quick and sure in your actions?	0.724	0.265	266	0.735	736	0.717	0.443	462	0.557	580		
Would you rate yourself as a lively individual?	0.779	0.232	233	0.768	770	0.792	0.256	261	0.744	759		
Would you be very unhappy if you were prevented from making numerous social contacts?	0.363	0.445	461	0.555	574	0.341	0.411	442	0.589	634		
Do you prefer action to planning for action?	0.306	0.318	313	0.682	672	0.328	0.342	351	0.658	676		
**NEUROTICISM 26**												
Do you sometimes feel happy, sometimes depressed, without any apparent reason?	0.720	0.506	528	0.494	515	0.656	0.205	226	0.795	876		
Does your mind often wander while you are trying to concentrate?	0.568	0.521	546	0.479	501	0.608	0.361	397	0.639	704		
Are you frequently lost in thought even when supposed to be taking part in a conversation?	0.566	0.584	609	0.416	433	0.613	0.572	625	0.428	468		
Are you sometimes bubbling over with energy and sometimes very sluggish?	0.619	0.410	424	0.590	610	0.586	0.269	293	0.731	795		
Are you inclined to be moody?	0.841	0.596	613	0.404	415	0.879	0.538	586	0.462	504		
Do you have frequent ups and downs in mood, either with or without apparent cause?	0.902	0.637	664	0.363	378	0.902	0.481	526	0.519	568		
		**Genotype A**		**Genotype G**			**Genotype AA**		**Genotype AG**		**Genotype GG**	
	**Loadings**	**%**	**Count**	**%**	**Count**	**Loadings**	**%**	**Count**	**%**	**Count**	**%**	**Count**
***MAOA* LATENT GENETIC FACTOR**												
RS3788862	0.989	0.703	816	0.297	344	0.968	0.524	618	0.392	462	0.085	100
RS5906957	0.989	0.763	885	0.237	275	0.968	0.604	713	0.344	406	0.052	61
RS979606	0.984	0.699	811	0.301	349	0.959	0.517	610	0.391	461	0.092	109

*Factor loadings were standardized estimates based on a multiple group model with strict measurement invariance constraints (Table 3, models m3 for males and f3 for females)*.

### Statistical analysis

**Figure 1:**
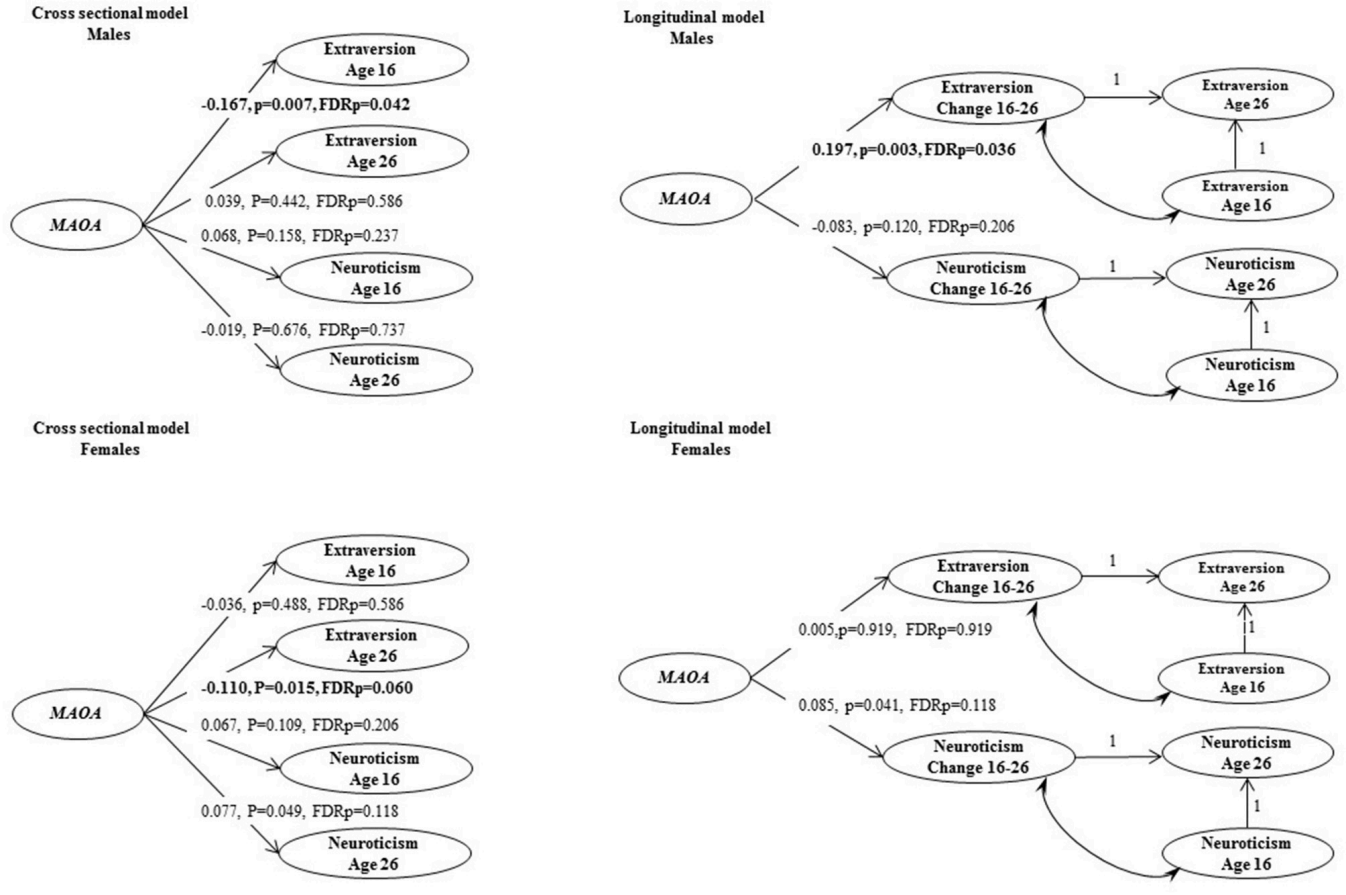
Cross-sectional and longitudinal models of genetic associations between *MAOA* and personality traits. Coefficients that are standardized bold values statistically significant at *P* < 0.05, after adjusting for false discovery rate.

The main analysis was based on a sample with complete genetic information, and at least one non-missing personality item at both ages. We first used longitudinal measurement invariance analyses to assess the measurement properties of the repeated personality measures. Then, the cross-sectional and longitudinal *MAOA* genetic effects on latent personality phenotypes were estimated using Structural Equation Models (SEM). For the cross-sectional analysis, *MAOA* latent genetic variable was specified to be a predictor of the latent variables representing personality traits (see Figure 1, cross-sectional model). For the longitudinal analysis, *MAOA* latent genetic variable was specified to be a predictor of the change in neuroticism and extraversion respectively (see Figure 1, longitudinal model).

#### Longitudinal measurement invariance analyses

Longitudinal measurement invariance analyses (Millsap, 2010) were conducted for the repeated dichotomous ratings of extraversion and neuroticism items in three steps: configural invariance (estimation of the same measurement model, with no added equality constraints), strong invariance (assessing the equality of factor loadings and item thresholds, which cannot be separated with binary rating scales) and strict invariance (assessing equality of factor loadings, item thresholds and residual variances). The goodness-of-fit of the models was evaluated using model fit indices. Since the chi-square is highly sensitive to sample size (Marsh et al., 1988, 2005), goodness of fit indices less sensitive to sample-size were also examined: the Root Mean Square Error of Approximation (RMSEA), the Tucker-Lewis Index (TLI), and the Comparative Fit Index (CFI; Fan et al., 1999; Hu and Bentler, 1999; Yu, 2002; Marsh et al., 2004). The TLI and CFI vary along a 0-to-1 continuum and values greater than 0.90 and 0.95 typically reflect an acceptable and excellent fit to the data. RMSEA values of less than 0.06 and 0.08 indicate a close fit and an acceptable fit to the data respectively. In terms of model comparisons for longitudinal measurement invariance analyses, a restrictive model is preferred if the change in model fit indices is not significantly inferior to those of the less restrictive model. For RMSEA, the change should be less than 0.015 (Chen, 2007). For CFI and TLI, the change should be less than 0.01 (Cheung and Rensvold, 2001; Chen, 2007). The chi-square difference tests compare the model under investigation to less restrictive alternative model and were computed with the DIFFTEST function for Mplus' robust weighted least square (WLSMV) estimator (Muthén and Muthén, 2015).

Since personality traits at ages 16 and 26 are repeated measures, we specified correlated residuals for the repeated items (McArdle, 2009).

#### Latent difference scores analyses

In order to assess the effect of *MAOA* gene on personality changes between age 16 and age 26, we derived factor scores of latent differences between age 16 and age 26 for extraversion and neuroticism, for males and females respectively. This is the longitudinal phenotype used in the current study and represents the change in personality from age 16 to age 26 (see Figure 1, longitudinal model). The use of latent different scores is valid in the circumstance that strong invariances are met as described in the previous section. This is because the calculation of latent differences scores requires estimation of factor means of repeated measures which is only meaningful when both factor loading and thresholds are invariant across both measurement occasions (McArdle and Prindle, 2008; McArdle, 2009). Technically in SEM framework, the latent difference score is represented by a latent variable that is obtained through regressing the time 2 latent variable on the time 1 latent variable, with the regression path set to 1 and the residual variances of the time 2 variable set to zero (see example Mplus syntax in Appendix). This way the time 2 variable is the sum of the time 1 variable and the latent change variable. In addition to the estimation of the variance of the latent difference score variable, latent mean of the change variable is also estimated, representing the increase/decrease of the latent variable since time 1, whose mean is usually set to zero as a baseline of comparison. Parameters of latent difference scores obtained this way are free of measurement errors which typically severely plagues the reliability of simple difference scores obtained from observed measures (Thomas and Zumbo, 2012; Kisbu-Sakarya et al., 2013).

#### Adjustment for multiple testing

To control for multiple testing issues in the main analysis, we applied false discovery rate (FDR; Benjamini and Hochberg, 1995) and reported adjusted *p*-values along with uncorrected *p*-values for evaluating statistical significance threshold of the genetic association tests.

#### Power analysis

We conducted both *post hoc* and *a priori* power analysis for the genetic latent variable predictor with statistically significant associations using statistical simulations. For *post hoc* power analysis, male sample size was fixed at 1,160, the same as in the analytic sample. Population parameters were also fixed to be equal to the sample data of the corresponding latent genetic variable and phenotype. For each power analysis, 1000 replication data sets were generated and analyzed, with results averaged across the 1000 analysis. The *a priori* power analysis was carried out with similar parameterization as the cross-sectional association model for extraversion at age 16 in males, for effect sizes with phenotypic variances explained at 1%, 2%, and 3%.

## Results

The descriptive statistics for the three *MAOA* SNPs is summarized in Table 1. All SNPs were in high linkage disequilibrium (LD), and with minor alleles exceeding frequency of 5%. The integrity of genotyping was checked by genotyping frequency, concordance of duplicates and Hardy-Weinberg equilibrium (HWE). The call rates for the genotyped SNPs were 97.8–99.2%, with >95% concordance between duplicate samples, and there was no evidence of deviation from HWE (in females only, *p* > 0.05). The *MAOA* latent variable was included in all subsequent analyses (factor loadings and genotype frequencies are presented in Table 2).

### Measurement invariance analysis of longitudinal phenotypes

The personality phenotypes (see Table 2 for item descriptions) were first subject to tests of longitudinal measurement invariance. Given that all measurement models included *MAOA* latent variables, these tests were conducted separately for males and females participants. In the configural model (Table 3, model m1 for males and model f1 for females), all factor loadings and thresholds were freely estimated with confirmatory factor analytic models. Results revealed that this model fitted the data very well. In the next model (model m2 for males and model f2 for females), both factor loadings and thresholds were constrained equal across time. Compared with the baseline model, changes in model fit indices remained minimal, thus supporting the strong longitudinal invariance of the model. In the third model (model m3 for males and model f3 for females), the residual variances of individual items were further constrained to equality across measurement waves. There were no changes in model fit indices for males, and there was even an improvement for females, supporting the strict measurement invariance of the personality measures across age 16 and 26, thus providing support for comparability of measurements across waves and further ground for using latent change scores.

**Table 3:** Model fit indices for the measurement invariance analyses of longitudinal phenotypes of personality traits and genetic association tests.

	**model**	***n***	**χ^2^**	**degree of freedom**	**free parameter**	**Model of comparison**	**χ2 Difference Test**	**p (degree of freedom)**	**RMSEA**	**CFI**	**TLI**
**MALES**											
Configural model	m1	1,160	808.683	303	75				0.038	0.991	0.989
Strong invariance	m2	1,160	878.578	311	67	m1	83.215	<0.001(8)	0.040	0.989	0.988
Strict invariance	m3	1,160	935.975	254	55	m2	63.882	<0.001(12)	0.040	0.989	0.988
SEM cross-sectional	m4	1,160	814.835	313	65				0.037	0.991	0.990
SEM longitudinal	m5	1,160	971.144	326	52				0.041	0.988	0.987
**FEMALES**											
Configural model	f1	1,180	1,054.311	303	78				0.046	0.986	0.984
Strong invariance	f2	1,180	1,116.822	311	70	f1	73.142	<0.001(8)	0.047	0.985	0.983
Strict invariance	f3	1,180	1,133.666	323	58	f2	32.860	<0.001(12)	0.046	0.985	0.984
SEM cross-sectional	f4	1,180	1,041.822	313	68				0.044	0.987	0.985
SEM longitudinal	f5	1,180	1,245.856	326	55				0.049	0.983	0.982

*RMSEA, Root Mean Square Error of Approximation; CFI, Comparative Fit Index; TLI, Tucker-Lewis Index*.

Factor loadings of models m3 and f3 are presented in Table 2, whereas correlations and latent difference means estimated from models m3 and f3 are presented in Table 4. In both male and female samples, strong correlations were observed between repeated extraversion (0.592 for males and 0.565 for females) and neuroticism (0.526 for males and 0.479 for males) factors, attesting to their stability over time. There were also weak to modest correlations between extraversion and neuroticism factors (−0.077 to −0.221 in males and −0.189 to −0.312 in females), attesting to their distinctiveness. Latent difference scores in extraversion and neuroticism were specified based on a re-parameterization of models m3 and f3. These latent difference factors are the longitudinal phenotypes used in genetic association analysis (Figure 1). The estimated means of the latent change scores were not statistically different from zero among the male and female samples (Table 4). However, the variances of the change scores were statistically significant, implying the presence of meaningful inter-individual variations in the change scores estimated for both samples.

**Table 4:** Factor means and correlations of extraversion and neuroticism in males (model m3) and females (model f3).

	**Correlation**					Latent difference^**^	
	**Extraversion 16**	**Extraversion 26**	**Neuroticism 16**	**Neuroticism 26**	***MAOA* factor**	Mean^*^	**Variances**
**MALES**							
Extraversion 16	1					0	NA
Extraversion 26	**0.592**	1				0.013	**0.243**
Neuroticism 16	−**0.220**	−0.077	1			0	NA
Neuroticism 26	−**0.221**	−**0.169**	**0.526**	1		**0.109**	**0.918**
*MAOA* factor	−**0.164**	0.040	0.068	−0.019	1	NA	NA
**FEMALES**							
Extraversion 16	1					0	NA
Extraversion 26	**0.565**	1				−0.023	**0.191**
Neuroticism 16	−**0.279**	−**0.189**	1			0	NA
Neuroticism 26	−**0.248**	−**0.312**	**0.479**	1		0.019	**0.720**
*MAOA* factor	−0.036	−**0.110**	0.067	**0.077**	1	NA	NA

*Bold values are statistically significant at P < 0.05*.

* *Means are based on original latent factor scales*.

** *Latent difference model is an identical model with alternative parameterization of the cross-sectional model (m3, f3)*.

### Genetic association analysis of cross-sectional and longitudinal phenotypes

Genetic association analyses were performed with the *MAOA* latent variable as a predictor in cross-sectional and longitudinal models of extraversion and neuroticism. SEM analyses demonstrated an association of the *MAOA* latent genetic variable (AAG) with lower extraversion scores at age 16 in males (Figure 1, β = −0.167, FDRp = 0.042). The *MAOA* latent genetic variable was also positively associated with change in extraversion in males (β = 0.197, FDRp = 0.036, see an example syntax in Appendix). No statistically significant associations between the *MAOA* gene and personality traits were found in females after multiple testing adjustment.

In an additional sensitivity analysis for the male participants, *MAOA* factor scores were calculated and categorized into high and low values groups, representing the top 30% and bottom 30% of the genetic scores respectively. Results from the additional analysis on this subsample (*n* = 695) showed larger effect sizes for both the association with age 16 extraversion (β = −0.264, *p* < 0.001), and the change in extraversion from age 16 to age 26 (β = 0.275, *p* < 0.001).

### Power analysis

To assess the statistical power of the analyses conducted in the male sample, Monte Carlo simulation analysis was performed both for our latent genetic approach and the traditional single SNP approach (Table 5). The latent genetic approach has a power of 81.2% for an effect size of 2.8% as observed in empirical cross-sectional association with extraversion at age 16. The power is 88.8% for an effect size of 3.9% as observed empirically for the change in extraversion from age 16 to age 26.

**Table 5:** Power analysis of latent genetic variable and single SNP approaches for SNPs with significant genetic effect in the male sample.

**Method**	**Latent gene approach**	**Single SNP approach**		
		**rs3788862**	**rs5906957**	**rs979606**
**CROSS-SECTIONAL ASSOCIATION WITH EXTRAVERSION AT AGE 16**				
Effect size	2.8%	1.3%	1.7%	1.5%
Power	81.2%	78.6%	74.5%	78.9%
**CHANGE IN EXTRAVERSION FROM AGE 16 TO AGE 26**				
Effect size	3.9%	1.8%	1.4%	2.5%
Power	88.8%	85.8%	84.2%	87.8%

*Effect size is based on percentage of explained phenotype variances by a single SNP predictor (i.e., the square of standardized regression coefficient in the case of a single predictor). Power statistic is based on 1,000 simulated replications; sample size was fixed at 1,160 consistent with the current male sample*.

We additionally conducted *a priori* power analysis with similar parameterization as the cross-sectional association model for extraversion at 16 in males, for effect sizes with phenotypic variances explained at 1%, 2%, and 3%. For 1% phenotypic variance explained, the resulted power was 0.55 based on latent genetic approach and 0.41 for the corresponding single SNP approach (with an effect size of 0.7%). For 2% phenotypic variance explained, the resulted power was 0.84 based on latent genetic approach and 0.71 for the corresponding single SNP approach (with an effect size of 1.2%). For 3% phenotypic variance explained, the resulted power was 0.95 based on latent genetic approach and 0.87 for the corresponding single SNP approach (with an effect size of 1.6%).

Both the post hoc and a priori power analysis have shown higher power in latent genetic approach compared to those found from single SNP approach, which will be even smaller once multiple testing corrections are applied for all three SNPs.

## Discussion

The association of the *MAOA* gene based on a latent genetic variable of three SNPs was investigated in relation to cross-sectional and longitudinal psychometric phenotypes of neuroticism and extraversion in a population-based study. The results revealed age-specific effects in males. In males, the *MAOA* latent genetic factor was associated with extraversion at age 16 and with the change scores in extraversion from 16 to 26 years.

Our findings for extraversion are in line with some previous studies of personality phenotypes. For example, the *MAOA* uVNTR is associated with Harm Avoidance (Yu et al., 2005), Persistence (Tsuchimine et al., 2008), Novelty Seeking and Reward Dependence (Shiraishi et al., 2006). Our study suggests that the three SNPs in the current investigation might function in similar fashion in terms of association with personality traits.

Another *MAOA* polymorphism, rs6323, located in exon 8 has a functional effect on mRNA level in the brain, with T allele associated with higher level and G allele with lower level of *MAOA* expression (Pinsonneault et al., 2006). One of our SNPs, rs979606, is in complete LD with rs6323, and the G allele of rs979606 is correspondent to the G allele of rs6323. As part of the latent genetic variable, the G allele of rs979606 was associated with lower extraversion scores in males in our study at age 16. This is in agreement with several studies suggesting that a *MAOA* variant with higher level of expression is associated with higher level of Novelty Seeking (Shiraishi et al., 2006), a phenotype closely related to extraversion (Zuckerman and Cloninger, 1996; De Fruyt et al., 2000; Livesley, 2001).

In general, the current study provides further evidence for the role of the *MAOA* gene in personality, and extraversion in particular. These genetic effects on extraversion may be underpinned by individual differences in brain structure and function. For instance, extraverts have more blood flow in the anterior cingulate gyrus, temporal lobes, and posterior thalamus, which are involved in sensory and emotional experience (Johnson et al., 1999) and can be affected by the *MAOA* genotype (Meyer-Lindenberg et al., 2006; Cerasa et al., 2010).

Our study showed more marked *MAOA* effect on extraversion in males than in females, indicating sex-specific association between the *MAOA* gene and personality (Deckert et al., 1999; Herman et al., 2005; Biederman et al., 2008). Genetic variants in the X-located *MAOA* gene may have different effects on cognition and behavior in males and females, and may explain sex differences in incidence and prevalence of certain psychopathologies. Indeed, several studies have found that various polymorphic variants in the *MAOA* gene (a 30-bp variable-number tandem repeat polymorphism in the promoter region; a GA repeat polymorphism in intron 2; and a G/T single-nucleotide polymorphism in exon 8) are associated with ADHD, ASD, and antisocial behavior, which have higher prevalence in men than in women (Karayiorgou et al., 1999; Huang et al., 2004). Furthermore, epigenetic regulatory mechanisms, such as methylation (Pinsonneault et al., 2006; Checknita et al., 2015) and incomplete X inactivation (Carrel and Willard, 2005), as well as regulation by Y-encoded transcription factor SRY (Wu et al., 2009) could contribute to sex differences of *MAOA*-related psychiatric disorders. These observed sex-specific associations might reflect sex differences in brain structure and function (Giedd et al., 1999; Cahill, 2009), but could also be due to uncertainties in the exact mechanisms as a result of X inactivation. For instance, one study found that the low expressed *MAOA* variant, associated with increased risk of violent behavior, was associated with changes in orbitofrontal volume, amygdala and hippocampus hyper-reactivity during aversive recall, and impaired cingulate activation during cognitive inhibition in men only (Meyer-Lindenberg et al., 2006). Nevertheless, it important that these results are interpreted with caution before the pathways of X inactivation are more thoroughly understood.

Our findings suggest that age is an important factor for effects of the *MAOA* gene on personality. In males, the *MAOA* gene was associated with extraversion at age 16, and with longitudinal change in extraversion between ages 16 and 26. This finding is consistent with other studies that suggest the dynamic nature of genetic effects. For example, it is known that genetic effects on some complex traits become stronger from childhood through adolescence and adulthood (e.g., Haworth et al., 2010; Gaysina et al., 2013). One of the plausible mechanisms of age-specific effects of genetic variants is the related changes in the gene expression (Francesconi and Lehner, 2014). It has been demonstrated that expression activity of many brain-expressed genes, including *MAOA* gene, can change during development and maturation both in human and animals (Vitalis et al., 2002; Naumova et al., 2013; Bakken et al., 2016). However, we should note that the evidence for the mechanisms of development-specific effects of MAOA remains limited. The data available on specific development stages refer mainly to the mammal brain at fetal and post-fetal stages (for review, see Nicotra et al., 2004). For example, one study used in situ hybridization and histochemistry to localize MAOA (and MAOB) in the developing nervous system of mice. This study found that during postnatal life, MAOA expression declines (Vitalis et al., 2002).

It is known that many developmental changes occur between ages 16 and 26 years, and young people are exposed to various influences from many environmental factors. Therefore, we cannot exclude possible gene-environment interactions effects on personality during the transition to adulthood. For example, several studies have reported an interaction between the *MAOA* gene and childhood stress on conduct and antisocial disorders, that is stronger in males (Kim-Cohen et al., 2006; Taylor and Kim-Cohen, 2007; Holz et al., 2014; Ouellet-Morin et al., 2016; Zhang et al., 2016). It may be the case that there are other environmental factors, such as significant life events, which interact with the *MAOA* gene and influence personality traits. This phenomenon may explain why genetic effects differ across the life course.

The present has a number of strengths and limitations. While most studies of *MAOA* gene effects on personality used small, cross-sectional samples, the present study has the advantage of using a population-based, longitudinal sample spanning from late adolescence to early adulthood. This developmental approach is of particular importance for studying the dynamic nature of genetic effects.

The application of latent variable methods is appropriate for modeling both the genetic variants and the phenotypes under investigation. Latent genotypic variables have previously been shown to be an effective way to capture genetic variances from multiple SNPs (Smyrnis et al., 2009; Tsonaka et al., 2012; Bentley et al., 2013). This approach summarizes genotypic information across all genetic variants studied and capturing potential correlations between them. Thus, the power of detecting genetic effects can be elevated compared with separate SNP testing which ignores this feature. The strengths and potential of this method in even larger sets of SNPs have been evaluated across several settings in Tsonaka et al. (2012). Genetic effects found in association studies of complex traits such as personality are usually small due to influences from multiple genes (Plomin et al., 1994), and gene-gene, gene-environment interactions (Mackay, 2001). Phenotypic measurement error is a possible contributor to inconsistent findings by attenuating the effect sizes (McCoach et al., 2007) and reducing statistical power to detect the genetic association with phenotypes (van der Sluis et al., 2010). The latent variable approach we applied is an effective approach to improve phenotype definitions (Smyrnis et al., 2009; Ducci et al., 2010; Gaysina et al., 2013; Xu et al., 2015).

Therefore, the methodological strength of the present study lies in the use of the latent variable approach which has several appealing features over other commonly applied methods. In particular, the analysis for each SNP separately is less powerful (as shown in both *a priori* and the *post hoc* power analysis) than the currently implemented latent variable method, because in single SNP analysis the number of tests depends on the number of SNPs in the gene-set of interest. Therefore, the multiple testing correction burden is greater. On the contrary, the latent variable method summarizes the genetic information from the SNPs via a single latent variable, therefore only a single test is implemented irrespective of the number of SNPs considered.

Another common practice used in studying multiple SNPs is the construction of polygenic scores. Although, this approach is computationally simpler (a sum of the SNPs weighted by the marginal effects of the SNPs on the phenotype) and easy to implement in practice, the polygenic score has two important limitations. First, it requires a priori knowledge of the separate SNP effects. Second, for its computation the sampling variability (i.e., standard error) of the separate SNP effects is ignored. This implies that the effects of the polygenic score will be inevitably attenuated (i.e., biased). This phenomenon has been thoroughly studied in the measurement error literature (e.g., Carroll et al., 2006). Our approach instead properly accounts for this sampling variability and does not require prior knowledge for the separate SNP effects as it provides an empirically derived optimal weighting of the different SNP scores.

The latent variable approach also provides stronger statistical power to detect statistically significant effects. The type I error and power of the latent variable approach have been thoroughly investigated empirically in the study of Tsonaka et al. (2012) where longitudinal genetic effects were studied. In this study, several scenarios were considered regarding the between-SNPs correlation structure (i.e., low vs. moderate correlation) including the size of the association of the subjects' genotypes with the phenotype, and size of the gene-set and sample size. The sample sizes considered there were much smaller than in the current study but the latent variable approach still achieves high and satisfactory levels of power while preserving the type I error at nominal level.

Furthermore, as shown in the additional simulation power analysis performed for the current study, the latent genetic approach demonstrated consistently higher statistical power compared to single SNP approach, even before applying corrections for multiple testing. Moreover, when analysing all the SNPs simultaneously in the same model, we explicitly took advantage of the potential correlations among SNPs. Both positive and negative correlations between the SNPs are allowed, while this correlation is totally ignored in the separate SNP tests. Although *post hoc* power analysis showed sufficient to high statistical power for the effect sizes observed in the current study, this is partly due to effect sizes which were relatively high compared to previous GWAS studies on gene based analysis (Luciano et al., 2017) or polygenic risk scores (van den Berg et al., 2016). The *a priori* power analysis showed that for studies with sample sizes comparable to the present investigation (*n* = 1,160), although the power (0.84) is sufficient for detecting variances explained at 2%, for effect size of 1% variance explained, the power drops to 0.41. Therefore, it is important to ensure the study is of sufficient sample size to detect expected effect sizes with sufficient power.

In terms of limitations, we did not formally test for population stratification, however all participants in the current study were of white Caucasian background, so the population stratification is unlikely. We did not take into account environmental exposures, whereas genetic factors and known to interact with environmental factors in their influences on complex traits. Similarly, in the current study, we did not have functional indicators (e.g., sex hormone levels) to test for effect modification that could correspond with developmental stages. Moreover, as already mentioned previously, our results in females need to be interpreted with caution. It is known that many X-chromosome genes may express differently in males and females, due to incomplete inactivation of one of two X chromosomes in females, as well as other mechanisms (as discussed above). In our study, information on the status of X-chromosome inactivation was not available. Future studies which incorporate such information would be very helpful in order to further clarify and determine the role of *MAOA* gene in personality traits. Furthermore, replications based on large studies are needed to confirm the effects observed in the male sample of the present study. As such, future GWAS studies could include genes on X chromosome both in male samples and in female samples for putative genetic associations.

In conclusion, the present study confirms that *MAOA* genetic variation affects personality traits in age-specific manner in males. Our study highlights the importance of applying a life course developmental approach to behavioral genetics (Scerif and Karmiloff-Smith, 2005). This is particularly important for candidate genes, such as *MAOA*, which is widely thought to interact with environmental factors to influence behavioral outcomes across developmental stages (Kim-Cohen et al., 2006). Future studies exploring the gene-environmental interaction in longitudinal design such as our study are likely to help clarify the role *MAOA* gene plays in the crafting of personality.

## Author contributions

MX, DG, and PJ designed the study, MX conducted analysis, MX and DG wrote up the manuscript; RT and JH contributed to the design of genotypic modeling; AM and TC contributed to design of phenotypic modeling. All authors contributed to the formulation of the study in its current form and improved iterative versions of the manuscript. All authors agree to be accountable for all aspects of the study and give final approval of the version to be published.

### Conflict of interest statement

The authors declare that the research was conducted in the absence of any commercial or financial relationships that could be construed as a potential conflict of interest.
