# Supplementary material for: Monoamine Oxidase A (MAOA) Gene and Personality Traits from Late Adolescence through Early Adulthood: A Latent Variable Investigation
Source: Front Psychol. 2017 Oct 11;8:1736. doi: 10.3389/fpsyg.2017.01736 (PMC5641687; doi:10.3389/fpsyg.2017.01736)
Supplement: Supplementary file 1 [file DataSheet1.docx]

**10 Appendix**

Mplus input syntax

TITLE: genetic association with latent change scores in males

DATA: FILE = "xxx.dat";

VARIABLE:

NAMES = sex rs3788862 rs5906957 rs979606 mpi1 mpi4 mpi5 mpi8 mpi9 mpi12

mpi2 mpi3 mpi6 mpi7 mpi10 mpi11 happ72 newf72 quick72 livey72 unhap72 plac72

hade72 wand72 lit72 ener72 updo72 mood72;

MISSING=.;

USEOBSERVATIONS ARE sex EQ 2;

USEVARIABLES ARE

rs3788862 rs5906957 rs979606

mpi1 mpi4 mpi5 mpi8 mpi9 mpi12 !extroversion variables age16

mpi2 mpi3 mpi6 mpi7 mpi10 mpi11 !neuroticism variables age 16

happ72 newf72 quick72 livey72 unhap72 plac72 ! extroversion variables age26

hade72 wand72 lit72 ener72 mood72 updo72; !neuroticism variables age26

CATEGORICAL ARE !declare variables as categorical

rs3788862 rs5906957 rs979606

mpi1 mpi4 mpi5 mpi8 mpi9 mpi12 !extroversion variables age16

mpi2 mpi3 mpi6 mpi7 mpi10 mpi11 !neuroticism variables age 16

happ72 newf72 quick72 livey72 unhap72 plac72 ! extroversion variables age26

hade72 wand72 lit72 ener72 mood72 updo72; !neuroticism variables age26

ANALYSIS:

ESTIMATOR IS wlsmv;

PARAMETERIZATION = THETA;

MODEL:

maoa by rs3788862@1 rs5906957 rs979606; !define *MAOA* gene variable

! define latent variable for personality phenotypes, with factor loading constrained equal across time

ext16 by mpi1 @1 mpi4 mpi5 mpi8 mpi9 mpi12 (e2-e6); !ext16

neu16 by mpi2 @1 mpi3 mpi6 mpi7 mpi10 mpi11 (n2-n6); !neu16

ext26 by happ72 @1 newf72 quick72 livey72 unhap72 plac72 (e2-e6); !ext26

neu26 by hade72 @1wand72 lit72 ener72 mood72 updo72 (n2-n6); !neu26

!constrain thresholds equal across time

[mpi2$1 hade72$1](int1);

[mpi3$1 wand72$1](int2);

[mpi6$1 lit72$1] (int3);

[mpi7$1 ener72$1](int4);

[mpi10$1 mood72$1](int5);

[mpi11$1 updo72$1](int6);

[mpi1$1 happ72$1](int7);

[mpi4$1 newf72$1](int8);

[mpi5$1 quick72$1](int9);

[mpi8$1 livey72$1](int10);

[mpi9$1 unhap72$1](int11);

[mpi12$1 plac72$1](int12);

!specify correlated residuals across time

mpi2 WITH hade72; mpi3 WITH wand72; mpi6 WITH lit72;

mpi7 WITH ener72; mpi10 WITH mood72; mpi11 WITH updo72;

mpi1 WITH happ72; mpi4 WITH newf72; mpi5 WITH quick72;

mpi8 WITH livey72; mpi9 WITH unhap72; mpi12 WITH plac72;

!constrain residual variances equal across time

mpi1-mpi11@1;

happ72-updo72@1;

!define latent difference score variables for extraversion

ext26@0;

ext26 ON ext16@1;

extlc by ext26 @1;

extlc*;

[extlc*];

extlc WITH ext16;

!define latent difference score variables for neuroticism

neu26@0;

neu26 ON neu16@1;

neulc by neu26 @1;

neulc*;

[neulc*];

neulc WITH neu16;

!fix latent means of personality phenotype to be 0

[ext16@0];

[neu16@0];

[ext26@0];

[neu26@0];

!Estimate association of *MAOA* with latent difference scores

extlc ON maoa;

neulc ON maoa;
